# Hot Trends in Pheochromocytoma and Paraganglioma: Are We Getting Closer to Personalized Dynamic Prognostication?

**DOI:** 10.5146/tjpath.2024.13681

**Published:** 2024-09-02

**Authors:** C. Christofer Juhlin, Ozgur Mete

**Affiliations:** Department of Oncology-Pathology, Karolinska Institutet, Stockholm, Sweden; Department of Pathology and Cancer Diagnostics, Karolinska University Hospital, Stockholm, Sweden; Department of Pathology, University Health Network, Toronto, ON, Canada; Endocrine Oncology Site, Princess Margaret Cancer Centre, Toronto, ON, Canada; Department of Laboratory Medicine and Pathobiology, University of Toronto, Toronto, ON, Canada

**Keywords:** Pheochromocytoma, Paraganglioma, Pathology, Diagnostic, Prognostic, Review

## Abstract

Pheochromocytoma and abdominal paraganglioma (PPGL) are rare catecholamine-producing, keratin-negative, non-epithelial neuroendocrine neoplasms characterized by a unique association with syndromic diseases caused by constitutional mutations in a wide range of susceptibility genes. While PPGLs are recognized for their malignant potential, the risk of metastatic disease varies depending on several clinical, histological, and genetic factors. Accurate diagnosis and prognosis of these tumors require a multidisciplinary approach, integrating insights from various medical specialties. Pathologists play a crucial role in this complex task, as numerous morphological, immunohistochemical, and genetic findings can be linked to worse outcomes. Therefore, it is vital to stay informed about the latest advancements in PPGL pathology. This brief review provides an overview of the challenges associated with PPGLs and highlights the most recent developments in tumor prognostication.

Pheochromocytomas and paragangliomas (PPGL) are non-epithelial neuroendocrine neoplasms of the paraganglia with unique hormonal, hereditary, and histological characteristics (1,2). Diagnosing these enigmatic neoplasms requires an integrated approach, including thorough patient history, symptomatology, biochemical analysis, radiology, histopathology, and genetic analysis, involving multiple specialties (3,4). The diagnosis is usually suggested following the identification of an adrenal or extra-adrenal mass on CT scan, followed by hormonal work-up identifying elevated levels of catecholamines (3-metoxytyramine/dopamine, normetanephrine/norepinephrine, and metanephrine/epinephrine) (5). From a histopathological perspective, PPGLs present challenges for surgical pathologists, as these tumors may mimic other lesions with similar growth patterns including epithelial neuroendocrine neoplasms of various sites (6,7). Therefore, care must be taken to verify the tumor using immunohistochemistry (1,8,9). By definition, PPGLs are keratin-negative, non-epithelial neuroendocrine neoplasms that show immunoreactivity to INSM1, chromogranin A and synaptophysin. They are usually positive for GATA3, and subsets of tumors may exhibit S100 nuclear and cytoplasmic expression. Moreover, recent data suggest that PHOX2B and Hand2 are reliable markers to distinguish PPGLs from epithelial neuroendocrine neoplasms (10,11). Since PPGLs are highly heritable neuroendocrine neoplasms, the standard care also includes the assessment of underlying germline susceptibility (1,12). While multifocality and some morphological findings can be used to support underlying germline susceptibility, immunohistochemical analysis related to pathogenic biomarkers can guide clinical genetics (e.g., SDHB, CAIX, alpha-inhibin, 2-SC, FH) (1). Moreover, in some circumstances, molecular immunohistochemistry, particularly SDHB or FH/2-SC can help to narrow down the pathogenicity of a variant of uncertain significance (VUS) (1,13).

It is now well-established that all PPGLs are malignant neoplasms with metastatic potential, but estimating the risk of metastasis is a difficult challenge. While multiparameter scoring systems are no longer parts of the routine pathology practice, how to properly estimate the risk of metastatic dissemination is a growing area of interest for multidisciplinary teams in endocrine oncology. The two most established histological algorithms for predicting aggressive biological behavior have limitations in reproducibility and positive predictive values (14). While these algorithms can potentially help to rule out tumors that are not at risk of spreading, they may also miss some tumors that subsequently metastasize. Even so, some adverse histological parameters seem important to recognize, such as the presence of angioinvasion, tumor necrosis, the overall growth pattern, cellularity, reduced to absent intratumoral sustentacular cell network, margin status and the extent of the tumor. As a result, modern endocrine oncology has now transitioned to a personalized dynamic risk stratification based on clinical/biochemical, pathological and molecular risk factors (Figure 1) (1).

**Figure 1:**
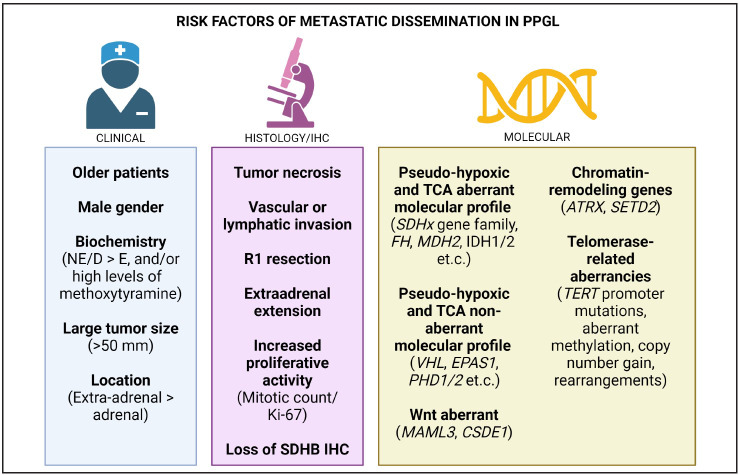
Clinical, histological, immunohistochemical and molecular approaches to identify pheochromocytoma and paraganglioma (PPGL) at risk of metastatic dissemination.

The underlying genetics can provide important clues with regards to risk of disease dissemination, as mutations in genes encoding various enzymes within the pseudo-hypoxia pathway regulation, especially tricarboxylic acid (TCA) cycle associated proteins, correlate to metastatic potential (9,15). Mutations in subunits of the *succinate dehydrogenase (SDH)* gene family, especially* SDHB*, *SDHC*, and* SDHD*, are notably associated with an increased risk of metastatic dissemination, making genetic analysis of prognostic value. Additionally, SDHB immunohistochemistry is a valuable tool for triaging cases for genetic screening, as SDHB immunoreactivity is lost in virtually all PPGLs driven by pathogenic *SDH*B, *SDHC* and *SDHD *variants (Figure 2). Similarly, alpha-inhibin immunohistochemistry has been shown to identify PPGLs with mutations in various genes within the pseudo-hypoxic pathway, not restricted to *SDHx* gene mutations (16). CAIX immunostaining may also identify *VHL*-mutated tumors, which carry a small but non-negligible risk of dissemination (17). Subsets of biologically aggressive PPGLs are associated with aberrant Wnt signaling, and these tumors often carry somatic *MAML3* gene fusions or *CSDE1* mutations (18). PPGLs in this cluster are more often metastatic than traditional PPGLs driven by kinase-related mutations.

**Figure 2:**
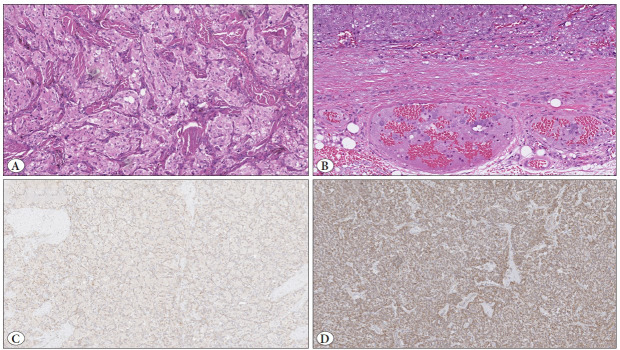
SDHB mutated pheochromocytomas and paragangliomas (PPGLs) have an increased risk of metastatic spread. **A)** Hematoxylineosin stained section of an SDHB mutated PPGL, displaying the characteristic eosinophilic cytoplasm with occasional vacuolization. **B)** Venous (vascular) invasion is often seen, a recognized adverse histological parameter. **C)** SDH deficiency on SDHB immunohistochemistry is characterized by loss of cytoplasmic granular reactivity in the tumor cells, with adjacent stromal and endothelial cells as internal positive control **D)** Positive SDHB staining in a PPGL with wildtype SDHx genes.

Although not coupled to adverse prognosis, the pathologist may facilitate detection of other gene mutations that may lead to the detection of hereditary disease. For example, rare cases of *MAX* mutated or *MAX* rearranged PPGLs typically exhibit loss of MAX protein expression, adding another potential tool to the diagnostic workup of these lesions. In contrast, there are no good commercial antibodies that can distinguish *NF1* and *RET-*mutant tumors (19,20). The assessment of non-tumorous parenchyma is critical since adrenal medullary hyperplasia is a sign of germline pathogenic variants (e.g., *RET, MAX, NF1, TMEM127, SDHB*) (1,21). The presence of composite tumor elements should raise the genetic screening of *RET, NF1, *and* MAX*-driven pathogenesis (1).

Years of focused PPGL research have also provided pathologists with better tools to assess the metastatic potential of these tumors, and many of the advances target proliferation markers or transcription factors associated with metastatic cases. The correlation between rapidly proliferating tumors and metastatic risk is well-known, and studies on the Ki-67 proliferation index in PPGL have shown higher proliferative activity in metastatic cases (9,22). Consequently, the two most established histologic algorithms for assessing proliferation risk in PPGL incorporate mitotic count or Ki-67 labeling index as weighted parameters (Figure 3). Similarly, the topoisomerase 2A (TOP2A) expression, a protein involved in chromosomal condensation and segregation during mitosis, is also increased in PPGLs with poor outcome (23). Overall, the proliferative activity seems to be an important factor when establishing the risk of dissemination, similar to other endocrine and neuroendocrine neoplasms.

**Figure 3:**
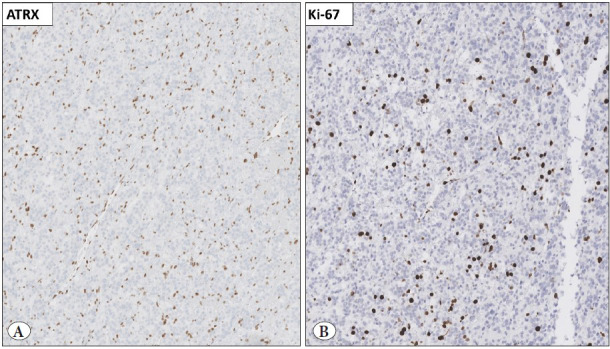
Immunohistochemistry in the triage of biologically aggressive aggressive pheochromocytomas and paragangliomas (PPGLs). **A)** ATRX immunohistochemistry can be used to identify underlying somatic ATRX gene mutations, a genetic event coupled to worse outcome. **B)** The Ki-67 labeling index is a reliable method to identify PPGLs with an increased

Moreover, somatic mutations in chromatin-remodeling and histone-modifying genes such as *ATRX, SETD2 *and* KMT2D* have been reported in metastatic PPGL (24,25). *TERT* gene aberrations, including promoter mutations, gene rearrangements, copy number gains, and aberrant methylation, have also been suggested as adverse molecular events (26–28). A recent study identified high mutational burden, microsatellite instability (MSI), increased somatic copy-number alterations, and *ATRX/TERT* aberrations as closely associated with metastatic disease (Figure 3) (29). Although many clinically accredited next-generation sequencing panels include *ATRX* and the* TERT* promoter, the combination of mutational burden, MSI, and gene copy number analyses is not yet used in clinical routine practice. However, the same study also identified CDK1 expression as highly associated with the aforementioned genetic profile in metastatic PPGL (29). Although promising from a clinical screening perspective, this observation needs to be verified by independent investigators.

Another area of increased focus in PPGL research is metabolomics and immune signatures (30,31). Mutations in TCA cycle components inhibit the metabolic pathway and lead to the accumulation of specific metabolites, including succinate, fumarate, and alpha-ketoglutarate, which can be oncogenic. Measuring these so-called onco-metabolites may have clinical value. Studies have shown that analyzing metabolite profiles can improve PPGL risk assessment, although the methodology remains expensive and rather intricate.

At the clinical and pathological level, distinction of multifocal primary disease from metastatic disease is an important challenge that requires knowledge of the distribution of the autonomous nervous system (1,2). As a result, tumors identified in liver and lungs are not always a sign of metastatic disease. The role of sustentacular cells in the distinction of metastatic disease from primary PPGL has also been evolved (32).

Even though histological, immunohistochemical, and molecular advances have increased our understanding of which PPGLs will subsequently disseminate, one must not overlook baseline clinical characteristics, which are important clues to identifying cases that will metastasize. For example, TCA-related PPGLs (with their inherent risk of dissemination) are overrepresented among cases with norepinephrine and/or dopamine production. Other established risk factors include large tumor size, older age at surgery, and male gender. These data are supported by recent machine learning models using artificial intelligence and clinical data from PPGL patients. These models showed that an algorithm considering plasma 3-methoxytyramine, metanephrine, normetanephrine, age, gender, previous history of PPGL, location and size of primary tumors, and the presence of multifocal disease was an efficient tool to predict metastatic disease (33). Thus, an integrated approach using clinical, pathological, and genetic variables is ultimately required to assess PPGL patients, and requiring well-established cooperation between radiologists, oncologists, geneticists, surgeons and pathologists (Figure 1).

## Conflict of Interest

The authors have no conflicts of interest to report.
